# Assessment of TiO_2_ Nanoparticle Impact on Surface Morphology of Chinese Hamster Ovary Cells

**DOI:** 10.3390/ma15134570

**Published:** 2022-06-29

**Authors:** Danute Batiuskaite, Ingrida Bruzaite, Valentinas Snitka, Arunas Ramanavicius

**Affiliations:** 1 Department of Biology, Faculty of Natural Sciences, Vytautas Magnus University, 58 K. Donelaicio Str., LT-44248 Kaunas, Lithuania; danute.batiuskaite@vdu.lt; 2 Department of Chemistry and Bioengineering, Faculty of Fundamental Sciences, Vilnius Gediminas Technical University, Sauletekio Av. 11, LT-10223 Vilnius, Lithuania; ingrida.bruzaite@vilniustech.lt; 3 Research Center for Microsystems and Nanotechnology, Kaunas University of Technology, 65 Studentu Str., LT-51369 Kaunas, Lithuania; vsnitka@ktu.lt; 4 Department of Physical Chemistry, Faculty of Chemistry and Geosciences, Vilnius University, 24 Naugarduko Str., LT-03225 Vilnius, Lithuania; 5 Laboratory of Nanotechnology, State Research Institute Centre for Physical Sciences and Technology, Sauletekio Av. 3, LT-10257 Vilnius, Lithuania

**Keywords:** TiO_2_ nanoparticles, nanoparticle–cell membrane interaction, atomic force microscopy, roughness, cells viability

## Abstract

The process of nanoparticles entering the cells of living organisms is an important step in understanding the influence of nanoparticles on biological processes. The interaction of nanoparticles with the cell membrane is the first step in the penetration of nanoparticles into cells; however, the penetration mechanism is not yet fully understood. This work reported the study of the interaction between TiO_2_ nanoparticles (TiO_2_-NPs) and Chinese hamster ovary (CHO) cells using an in vitro model. The characterization of crystalline phases of TiO_2_ NPs was evaluated by transmission electron microscopy (TEM), X-ray diffraction (XRD) spectrum, and atomic force microscopy (AFM). Interaction of these TiO_2_ nanoparticles (TiO_2_- NPs) with the CHO cell membrane was investigated using atomic force microscopy (AFM) and Raman spectroscopy. The XRD analysis result showed that the structure of the TiO_2_ particles was in the rutile phase with a crystallite size of 60 nm, while the AFM result showed that the particle size distribution had two peaks with 12.1 nm and 60.5 nm. The TEM analysis confirmed the rutile phase of TiO_2_ powder. Our study showed that exposure of CHO cells to TiO_2_-NPs caused morphological changes in the cell membranes and influenced the viability of cells. The TiO_2_-NPs impacted the cell membrane surface; images obtained by AFM revealed an ‘ultra structure‘ with increased roughness and pits on the surface of the membrane. The depth of the pits varied in the range of 40–80 nm. The maximal depth of the pits after the treatment with TiO_2_-NPs was 100% higher than the control values. It is assumed that these pits were caveolae participating in the endocytosis of TiO_2_-NPs. The research results suggest that the higher maximal depth of the pits after the exposure of TiO_2_-NPs was determined by the interaction of these TiO_2_-NPs with the cell’s plasma membrane. Moreover, some of pits may have been due to plasma membrane damage (hole) caused by the interaction of TiO_2_-NPs with membrane constituents. The analysis of AFM images demonstrated that the membrane roughness was increased with exposure time of the cells to TiO_2_-NPs dose. The average roughness after the treatment for 60 min with TiO_2_-NPs increased from 40 nm to 78 nm. The investigation of the membrane by Raman spectroscopy enabled us to conclude that TiO_2_-NPs interacted with cell proteins, modified their conformation, and potentially influenced the structural damage of the plasma membrane.

## 1. Introduction

Titanium dioxide (TiO_2_) is widely used as a pigment in cosmetics, the food industry, sunscreen production, and sensorics [1,2,3]. The production of TiO_2_ nanoparticles is growing, and it currently stands at millions of metric tons [4]. Due to the extensive use of TiO_2_ nanoparticles, they are widely distributed in the daily life of the human population and the environment in general. Under various conditions, titanium can form oxides with different oxidation states, which possess great catalytic properties [5]. Therefore, TiO_2_ nanoparticles constantly affect human and animal bodies. However, the interaction of TiO_2_-NPs with cells and their toxicity toward an epithelial cell line are still poorly investigated. TiO_2_-NPs interact with some phospholipids through electrostatic and non-electrostatic interactions [6]. It is also known that TiO_2_-NPs may enter inside cells [7,8]. Internalization of TiO_2_-NPs occurs via endocytosis-based mechanisms [9]. The toxicity of TiO_2_-NPs has been studied in vitro and in vivo [10,11,12,13]. However, so far, not all important aspects of the interaction of TiO_2_-NPs with living cells are clear because the interaction of TiO_2_-NPs with cells and the toxicity of nanoparticles depend on many factors, such as the type of cell and the size and shape of TiO_2_-NPs. One of the important and unclear questions is the influence of TiO_2_-NPs on the plasma membranes of cells [14]. Very few data have been published on the influence of nanoparticles on the membrane morphology and roughness. The application of AFM to study the CHO membrane morphology at the nanometer level and to apply this methodology to study the influence of TiO_2_-NPs on the destruction of the membrane constitute novel research.

Therefore, it is important to investigate the influence of TiO_2_-NPs on plasma membranes because we hypothesize that TiO_2_-NPs would affect some characteristics of the membranes, as well as influence changes in cells physiology or even induce some apoptotic effects. The Chinese hamster ovary (CHO) cell line is widely used as a model in many investigations, and the methods of cell growth are well known.

This work aimed to assess the influence of TiO_2_ nanoparticles (TiO_2_-NPs) on CHO cells, evaluate the interaction of TiO_2_-NPs with the CHO cell membrane using AFM and Raman spectroscopy, and assess the influence of TiO_2_-NPs on cell membrane integrity and the viability of CHO cells.

## 2. Materials and Methods

### 2.1. Characterization of TiO_2_ Nanoparticles

TiO_2_ nanoparticles were obtained from Nanostructured and Amorphous Materials Inc., Los Alamos, NM, USA. The composition and structure of TiO_2_ nanoparticles were characterized by X-ray diffraction (XRD), transmission electron microscopy (TEM), high-resolution field-emission scanning electron microscopy (FESEM), and Raman spectroscopy. The XRD measurements were performed on a SmartLab X-ray Diffraction System, Rigaku Corporation, Tokyo, Japan (Cu/45 kV/200 mA; duration/scan speed: 1°/min; step/sampling step: 0.01°; measurement axis: 2θ; scan range: 20–73°. For the measurements, we used compressed TiO_2_ powder on a glass substrate.

The TEM analyses were performed using a JEOL JEM 2010F electron microscope operating at an accelerating voltage of 200 kV, with a point-to-point resolution of 0.19 nm. The microscope was equipped with a Gatan electron energy loss imaging filter with an energy resolution equal to 0.7 eV and a Multiscan CCD 1 k × 1 k CCD camera for image acquisition. It was also equipped with an Oxford INCA ENERGY 300 energy-dispersive X-ray spectrometer (EDS, Oxford Instruments Analytical, High Wycombe, UK). The FESEM measurements were performed using an FEI Nova NanoSEM 630 FEG (FEI, Eindhoven, The Netherlands). Two preparation methods were used: dry powder compress on a Si wafer, and deposition of 10 µL of a 1% solution of TiO_2_ in water on a Si wafer before drying 1 h at 60 °C.

Dispersions were made at 3.24 mg/mL in Nanopure water using 2 wt.% serum with 16 min of sonication on an ice bath. Deposition on the Si wafer was achieved by spinning.

The Raman investigations were performed on the NT-MDT Ntegra Spectra system software version Nova 1.1.0.1844, NT-MDT Inc. Appendorn, The Netherlands using an inverted configuration (with a 100× high-NA objective, TE-cooled (down to −60 °C) CCD camera) at an excitation wavelength of 632.8 nm and a controlling laser power of 70 mW (acquisition time: 30 s). The drop (10 µL) of TiO_2_ water solution (1 mg/1 mL) was placed onto the glass substrate (Carl Roth, 50 × 24 mm, #1), and then dried and measured.

Characterization of TiO_2_-NP size was performed using atomic force microscopy (AFM) with Solver equipment from NT-MDT Inc. (Appendorn, The Netherlands) in tapping mode using commercial silicon cantilevers NSG11 with a force constant of 5 N·m^−1^. The TiO_2_-NP water solution (of 1 mg/1 mL) was filtered through a 200 nm polyethersulfone pore membrane Chromafil PES-20/25, purchased from Macherey-Nagel (Dueren, Germany), placed onto a glass substrate of 50 × 24 mm, #1 from Carl Roth, and then dried and assessed. Image analysis, data processing, and image acquisition were performed using NOVA software from NT-MDT Inc.

### 2.2. Cell Culture and Sample Preparation

The cells we used in the present work were from the Chinese hamster ovary (CHO) cell line. The culture medium was Dulbecco’s modified Eagle medium (DMEM) (D5546, Sigma-Aldrich, Chemie GmbH, Steinheim, Germany) with 10% fetal bovine serum (FBS), 1% l-glutamine solution, 90 μg/mL streptomycin, and 100 U/mL penicillin. First, 100 µL of the cell suspension (1 × 10^6^ cells/mL) was seeded on the surface of a silicon plate into Petri dishes (40 mm diameter) filled with 2 mL of the culture medium. The cells were incubated for 24 h to reach a confluent cell monolayer. The TiO_2_-NP dispersion was sonicated at 37 kHz ultrasound frequency in an Elma S30H Elmasonic Ultrasonic Cleaner from Elma GmbH & Co KG, (Singen, Germany) for 15 min. The culture medium was removed from Petri dishes with cells, which were washed with PBS, pH 7.4; lastly, Petri dishes were filled with PBS solution. After that, 50 µg/mL of TiO_2_-NPs were added to the Petri dishes with the cells. The cells were incubated for 20, 40, or 60 min. After the appropriate duration of incubation, PBS was aspirated from the cells, and the cells were washed with PBS, fixed with 4% formaldehyde, dissolved in PBS for 10 min at room temperature, washed with deionized water, and dried in air. Control samples were prepared under the same conditions except for the treatment of TiO_2_-NPs. The viability of CHO cells was evaluated by a colony formation assay [15]. Petri dishes with a diameter of 40 mm were filled with 2 mL of complete DMEM, which was then supplemented with 50.0 μg/mL of TiO_2_. Control samples (without TiO_2_) of CHO cells with 0.9% NaCl solution were prepared. The Petri dishes were incubated for 30 min under the same conditions as described above. Approximately 300 cells per Petri dish were seeded. Then, the CHO cells were incubated for 5 days for the formation of colonies. After the incubation, the cell colonies were fixed using 70% ethanol. After fixation, the cells were stained using Gram’s crystal violet solution 9 (Gram’s crystal violet solution—from Fluka Chemie (Buchs, Germany), air-dried, and counted through a binocular light microscope with 16× magnification. The viability of experimental groups was expressed as a percentage of colonies compared to control (100%). Digital images of the colonies were taken using a Moticam 2300 camera (Motic, Hong Kong) connected to the inverted ECLIPSE TS100 microscope (Nikon, Japan) at 40 × magnification.

### 2.3. Sample Analysis

The topography of the CHO cells was evaluated by AFM imaging, and measurements of the cells’ surface roughness were performed using the NT-MDT Solver system from NT-MDT Inc. in semi-contact mode using commercial silicon cantilevers with a tip diameter of 10 nm and force constant of 1.5 N/m. Cell surface roughness analysis was performed on the topography images of cell surface areas (5 × 5 µm) using Nova software from NT-MDT Inc. Surface roughness factors (10-point height (Sz) and average) were determined.

Energy-dispersive X-ray spectroscopy (EDS) analysis was performed to determine the TiO_2_-NPs content in CHO cells after exposure to the TiO_2_-NP colloidal solution. The samples were examined using a Hitachi S-3400N Type II scanning electron microscope (Tokyo, Japan).

The Raman spectra of CHO cells were registered using a confocal Raman system ‘upright INTEGRA Spectra’ from NT-MDT, using a 100× objective, 20 mW 532 nm wavelength DPSS laser, and a spectrometer—Solar TII from NT-MDT, equipped with a TE-cooled (−60 °C) CCD camera—DV401-BV from Andor Technology (Oxford Instruments, Abingdon, UK). The power of the laser at the sample was 0.4 mW, and the acquisition time was 20 s.

## 3. Results and Discussion

### 3.1. Analysis of the Structure of TiO_2_ Nanoparticles

#### 3.1.1. XRD Diffraction

**Figure 1:**
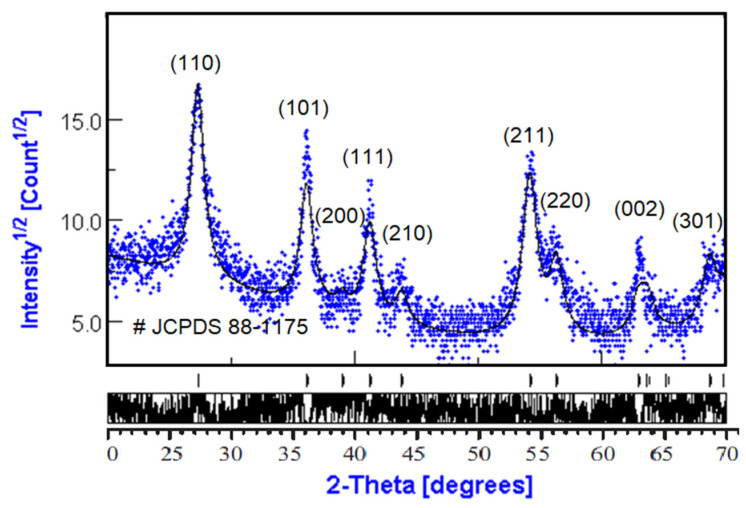
X-ray diffraction of rutile TiO_2_ nanopowders.

The phase composition and average crystallite size were obtained from the diffraction pattern presented in Figure 1. Diffraction patterns of rutile TiO_2_ powders were compared regarding the JCPDS database.

Analysis of XRD data was performed using MAUD software (Material Analysis Using Diffraction version 2.97, Radiographema, University of Trento, Trento, Italy, http://maud.radiographema.eu/, accessed on 22 May 2022). This is a general diffraction/reflectivity analysis program mainly based on the Rietveld method and whole-profile pattern fitting. The Rietveld method was selected for analysis because of the nanopowder structure of TiO_2_ samples (Table 1). The best fitting was obtained in the isotropic model of nanocrystals with a size of 10.5 ± 0.2 nm; the phase composition was 100% rutile TiO_2_ (Table 2).

The manufacturer’s data sheet of TiO_2_ rutile for comparison with the obtained experimental data is presented in Table 3. The experimental nanoparticle size and structure were in good agreement with manufacturer data.

#### 3.1.2. Transmission Electron Microscopy (TEM) and FESEM

**Figure 2:**
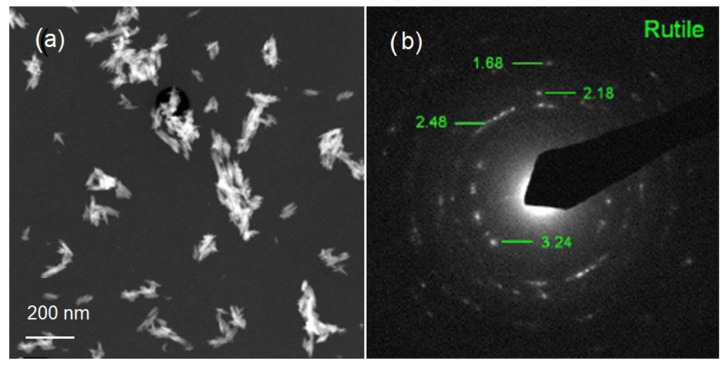
Images of the rutile phase: (**a**) TEM image of nano-TiO_2_ powder; (**b**) TEM image of nano-TiO_2_.

**Figure 3:**
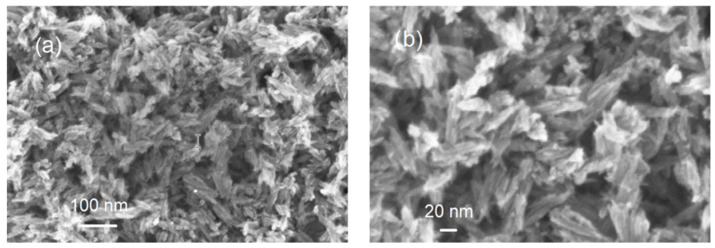
Images of FE scanning electron microscopy: (**a**) scale bar = 100 nm; (**b**) scale bar = 20 nm.

TEM was used to further examine the particle size, crystallinity, and morphology of the samples. TEM field images of TiO_2_ nanopowders in the rutile phase are shown in Figure 2a. It can be estimated that the particle size of powders in Figure 2a was nanoscale with a grain size of about 10 nm. The corresponding selected area electron diffraction (SAED) patterns of nano-TiO_2_ powders in the rutile phase are shown in Figure 2b. These are in agreement with the XRD results in Figure 1. In Figure 2b, the selected area electron diffraction (SAED) patterns of nano-TiO_2_ powders in the rutile phase showed spotty ring patterns without any additional diffraction spots and rings of second phases, revealing their crystalline structure. The size, shape, and surface morphology of the rutile TiO_2_ materials were studied using FESEM, and the images are presented in Figure 3. The rutile TiO_2_ was found to possess submicron particles consisting of a few fused needle-shaped nanoparticles. The nano-resolution imaging of these particles revealed the presence of nanocrystalline domains, corresponding with data of other authors [16,17,18].

From the FESEM results, it can be concluded that the TiO_2_ nanoparticles had a size of about a few tens of nanometers.

#### 3.1.3. Raman Spectroscopy Analysis

**Figure 4:**
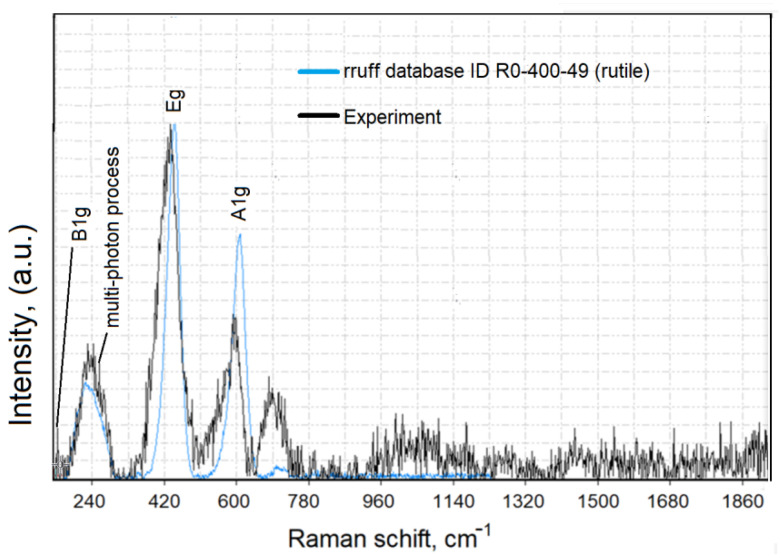
Raman spectra of rutile TiO_2_.

The Raman spectra of rutile TiO_2_ powder are presented in Figure 4.

**Figure 5:**
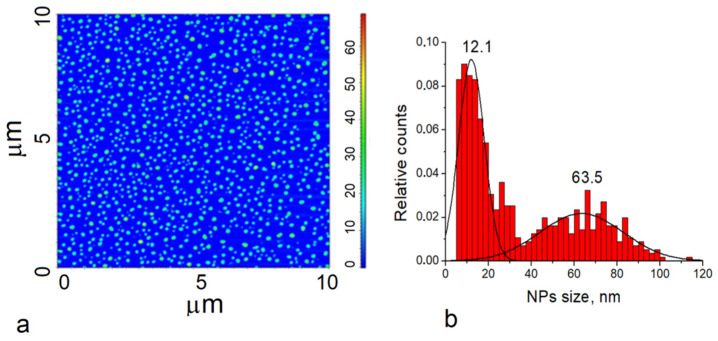
Atomic force microscopy-based analysis of TiO_2_-NPs (rutile) solution: (**a**) phase image of TiO_2_-155 NPs; (**b**) size distribution of TiO_2_-NPs (rutile).

For the removal of the background of the Raman spectra and a comparison of the measured data with literature data (RRUFF Project database, http://rruff.info/, accessed on 25 May 2022), we used CrystalSleuth software (http://rruff.info/about/about_download.php, accessed on 25 May 2022). The Raman spectrum of TiO_2_ (black line) correlated well with the RRUFF data (blue line). The Raman spectroscopy results (Figure 4) confirmed that the typical structure of titanium oxide was rutile in the tetragonal space group. Rutile has four Raman active modes, B_1g_, B_2g_, E_g,_ and A_1g._ The bulk Raman frequencies of the rutile phase are at 143 (B_1g_), 447 (E_g_), 612 (A_1g_), and 826 (B_1g_) cm^−1^. In a perfect infinite crystal, only phonons close to the center of the Brillouin zone (BZ) contribute to inelastic scatterings of incident radiations. When crystal sizes range in the nanometer scale, a larger portion of the BZ is allowed to effectively participate in scattering processes due to the weakening of the selection rule at q_0_  ≈  0. Therefore, a variation in the Raman frequency peaks and the shape of the Raman band can be observed. To determine the shape and size of the TiO_2_-NPs (rutile) used in the present work, the samples were imaged using an atomic force microscope. Figure 5 shows the size analysis results of TiO_2_-NPs (rutile).

#### 3.1.4. AFM Measurements of TiO_2_ Nanoparticle Size

The shape of the TiO_2_-NPs was determined using the data analysis of AFM measurements. The phase image of TiO_2_-NPs presented spherical nanoparticles with lighter and darker areas (Figure 5a). By contrast, the 10 μm × 10 μm topographic image roughness analysis of the TiO_2_-NPs showed that some of the particles were of different heights. TiO_2_-NPs were found to range in size from 5 nm to 60 nm.

### 3.2. Analysis of CHO Cell Topography and Surface Roughness

**Figure 6:**
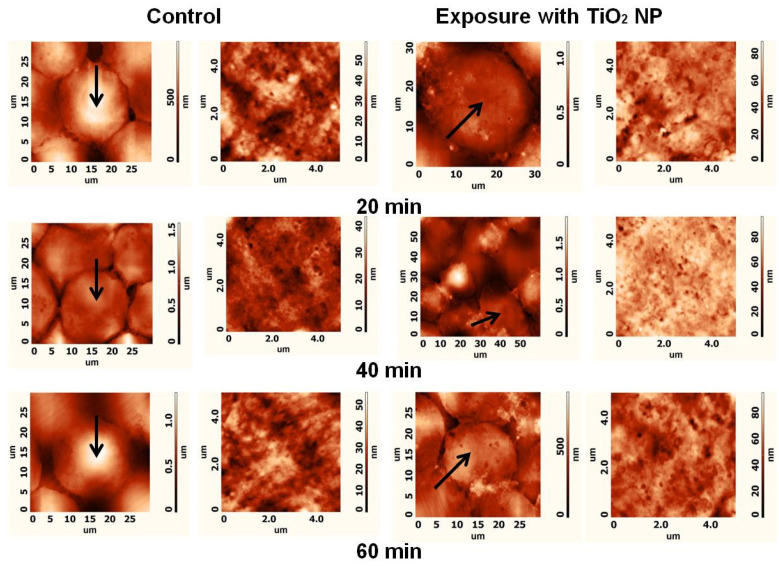
Representative AFM images of CHO control cells and cells after 20, 40, or 60 min exposure to TiO_2_; the first and the second columns show images of the topography of the whole cell and cell plasma membrane ultrastructure (5 × 5 µm), respectively, in the cases of the control; the third and the fourth columns show images of the topography of the whole cell and cell plasma membrane ultrastructure (5 × 5 µm), respectively, after exposure to TiO_2_-NPs. Investigated cells are indicated by arrows in the whole-cell images of the topography.

The aim of this in vitro study was to evaluate the direct impact of titanium oxide nanoparticles on the morphology and functionality of CHO cells to analyze how this specific cell type is influenced by oxidative TiO_2_-NPs. To estimate the impact of TiO_2_-NPs on CHO cell plasma membranes, CHO cells were treated with the TiO_2_-NP solution in PBS for 20, 40, or 60 min. Figure 6 shows representative AFM images of the cell membrane topography of the CHO cells. The areas with a different plasma membrane ‘ultrastructure’ were evaluated as surface height differences, and pits were observed in the images of the cell’s plasma membrane. It can be assumed that these pits were caveolae [19] participating in the endocytosis of TiO_2_-NPs.

**Figure 7:**
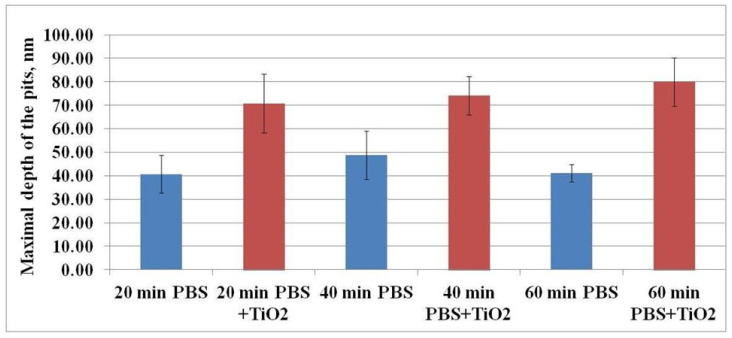
The dependence of the maximal depth of the pits on the exposure time of CHO cells to TiO_2_-NPs.

Figure 7 shows the dependence of the maximal depth of the pits on the exposure time of CHO cells to TiO_2_-NPs when compared to the control level. The maximal depth of the pits after exposure to TiO_2_-NPs for 20, 40, and 60 min was 70.73 ± 12.53, 74.07 ± 8.13, and 79.92 ± 10.41 nm, whereas, in the case of the control sample, these values were 40.61 ± 8.05, 48.82 ± 10.25, and 41.17 ± 3.70 nm, respectively. Hence, the maximal depth of the pits after the treatment with TiO_2_-NPs was 100% higher than the control values.

The results suggest that the higher maximal depth of the pits after exposure to TiO_2_-NPs was determined by the interaction of these TiO_2_-NPs with the cell’s plasma membrane. It is known that one of the functions of caveolae is endocytosis [20], and nanoparticles can enter the cells in this way [21,22,23]. Hence, it can be assumed that, in our study, TiO_2_-NPs were able to enter the cells via endocytosis. Moreover, these pits (or the part of them) may indicate plasma membrane damage (hole) caused by TiO_2_-NPs.

**Figure 8:**
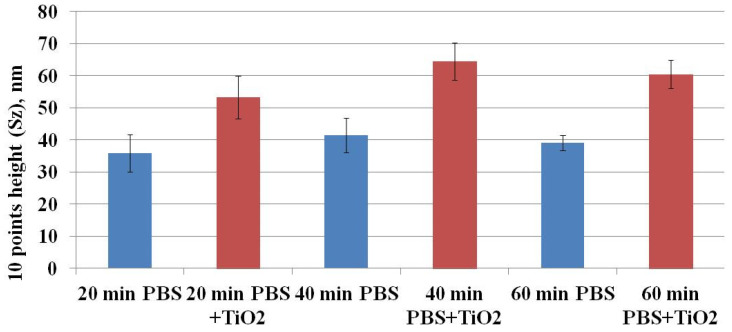
The dependence of the 10-point height (*S*z) surface roughness factor on the exposure time of CHO cells to TiO_2_-NPs.

**Figure 9:**
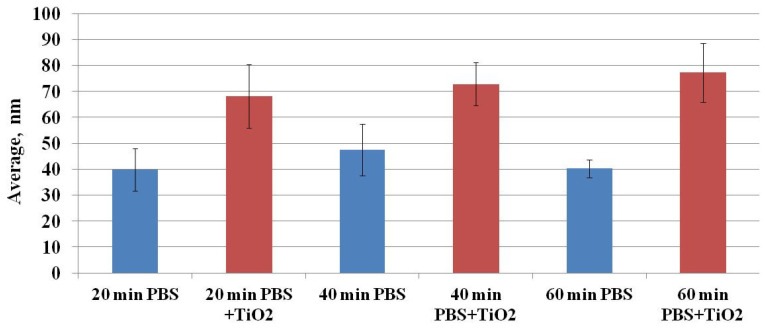
The dependence of the average surface roughness factor on the exposure time of CHO cells to TiO_2_-NPs.

Ruenraroengsak and coauthors analyzed live transformed human alveolar epithelial type 1-like cells exposed to amine-modified polystyrene latex nanoparticles (NPs) NPs using hopping probe ion conductance microscopy and observed severe damage and holes on cell membranes [24,25]. To quantitatively assess the CHO cell’s surface changes, surface roughness analysis was conducted. Surface roughness factors (10-point height (*S*z) and average) were determined. Figure 8 shows the results of *S*z factor determination. The *S*z factor after CHO cell exposure to TiO_2_-NPs for 20, 40, or 60 min was higher compared to the control (after exposure to TiO_2_-NPs—53.22 ± 6.60, 64.36 ± 5.78, and 60.39 ± 4.37 nm vs. control—35.92 ± 5.82, 41.43 ± 5.47 and 39.10 ± 2.32 nm, respectively) (Figure 8). A similar tendency was observed for estimation of the average (Figure 9) factor. The results suggest that changes in cell surface roughness were influenced by the impact of TiO_2_-NPs.

EDS and Raman spectroscopy analyses were performed to confirm that the changes in the CHO cell membrane roughness were caused by the interaction of the TiO_2_-NPs with the cell membrane. EDS analysis was performed to evaluate the content of TiO_2_-NPs in CHO cells. The Raman laser beam during the measurement was focused inside the cells to get a signal from TiO_2_-NPs, which had passed the membrane and accumulated in the cells.

**Figure 10:**
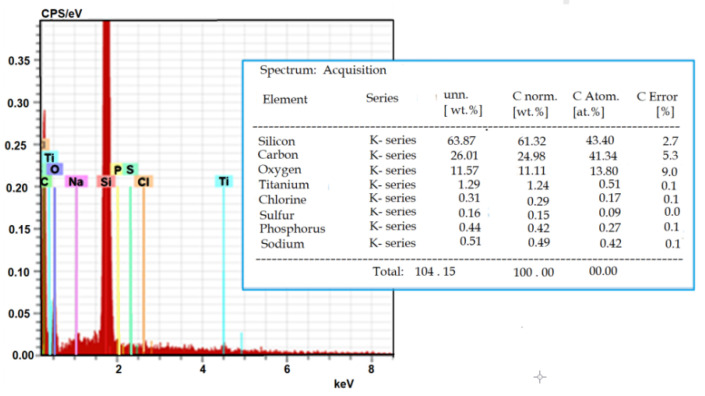
Representative EDS spectrum and elemental composition of the elements detected by EDS in CHO cells.

Figure 10 shows the results of the EDS analysis. It was established that TiO_2_-NPs were accumulated inside the CHO cells (Ti constituted 0.51% of the total observed elements). The high value of Si was a consequence of the used silicon substrate.

**Figure 11:**
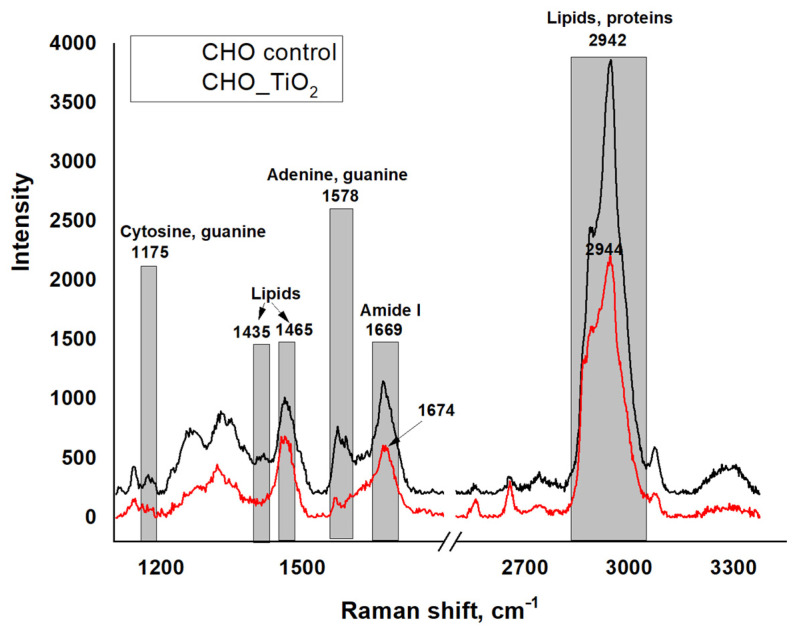
Raman spectra of CHO cells for control vs. after TiO_2_-NP treatment.

The investigation of the interaction of TiO_2_-NPs with the CHO cells was achieved using confocal Raman spectroscopy. We hypothesized that titanium dioxide nanoparticles interacted with lipid and/or protein moieties in the cell membrane. As can be seen in Figure 11, in the control case (black spectral line), a low-intensity peak was observed at 1435 cm^−1^, which is characteristic of C–H vibration in lipids. Following the release of the TiO_2_-NP suspension in the cell-containing sample, the peak disappeared in this area, indicating that the lipid membrane was deformed due to the interaction with TiO_2_-NPs [20]. The TiO_2_-NPs interacted with proteins and lipids, showing an intense peak in the Raman spectrum at 2942–2944 cm^−1^. This vibrational region is classified as the oscillation of aromatic and aliphatic amino acids, but it overlaps with characteristic C–H vibrations from fatty acids [26,27]. As seen from the Raman spectra, the control and test specimens differed in the shape of the lipid/protein domains, suggesting that this change was influenced by the interaction of TiO_2_-NPs with proteins and/or lipids in the cell membranes. This interaction is also supported by the results of atomic force microscopy, which clearly showed that the membrane was fragmented after the exposure of the cells toTiO_2_-NPs.

Another important area of the Raman spectrum characterizing the interaction of TiO_2_-NPs with living cells is in the 1669–1674 cm^−1^ interval. This is the vibrational region of amide I, which originates from peptide bonds and characterizes the secondary protein structure. As seen from the control spectrum, the amide I band was recorded at 1669 cm^−1^, indicating the dominance of α-helices in the protein structure. Meanwhile, the region characterizing amides in the cells exposed to nanoparticles shifted toward a longer wavelength (1674 cm^−1^), indicating the dominance of β-fibrous proteins after exposure to TiO_2_-NPs. These changes enable us to conclude that TiO_2_-NPs interacted with cell proteins, modified their conformation, and potentially influenced the structural damage of the plasma membrane, thus stimulating cytotoxicity.

### 3.3. Cytotoxicity and Cell Viability

**Figure 12:**
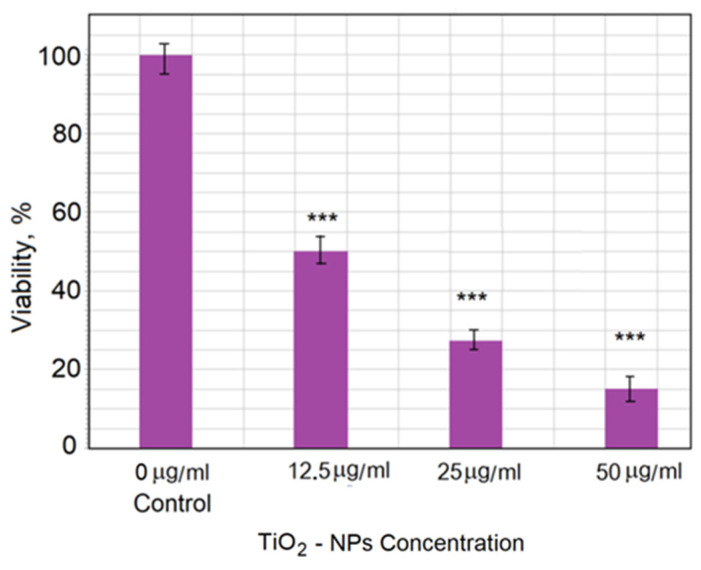
Viability of CHO cells after treatment with TiO_2_-NPs at different concentrations. The data are expressed as the mean ± standard error (SE) from three independent experiments. Statistical analysis was performed using Student’s *t*-test (*** *p* < 0.005). All data for TiO_2_-NP treatment were statistically significantly different from the control (*p* < 0.005) (marks are not displayed in the histogram), as well as between 12.5 μg/mL TiO_2_-NP and 50.0 μg/mL TiO_2_-NP treatments (*p* < 0.005).

The TiO_2_-NP cytotoxicity toward CHO cells was analyzed over 5 days of exposure. Figure 12 shows the relationship between cell viability (as a percentage of control value) and TiO_2_-NP concentration. The strongest effect was observed for 50 μg/mL TiO_2_, which showed an 85% decrease in viability compared with the control group.

Data show that the viability of cells decreased with increasing concentration of TiO_2_-NPs. The obtained AFM images of cell topography and the detailed cell morphology and roughness investigation demonstrated the changes after the cell membrane interaction with nanoparticles. This study showed that the surface roughness and the number and size of pits in the membrane changed depending on the time of incubation. This may have been a result of the restructuring of the membrane during endocytosis or adaptation of the membrane in response to the interaction with TiO_2_-NPs.

Da Rosa, using scanning electron microscopy and force spectroscopy, demonstrated that TiO_2_-NP aggregates influenced morphological changes and stronger stiffness features in neutrophils after 1 min of exposure. Furthermore, neutrophils retained higher elasticity for a long time, possibly due to intense phagocytosis and cell stiffness following tip indentation. SEM images suggested an alteration of cell morphology over time probably related to activation, cytoskeleton rearrangement, and phagocytosis [28].

Pasold and coauthors investigated the influence of titanium and zirconia particles on the morphology and functionality of mature human osteoclasts [29]. It was found that the bioreactivity, including cytotoxicity, of nanoparticles depended both on the particle amount and characteristics (type of material, size, morphology, concentration, etc.) and on the susceptibility of the endoprosthetic patient. The morphological change in cellular apoptosis initiated by the change in membrane roughness was investigated using AFM by Wang and coauthors [30]. The mouse monocyte/macrophage cell line RAW 264.7 was subjected to apoptotic induction by hydrogen peroxide. To identify cellular apoptosis in its early stage, an atomic force microscope was adapted to thoroughly reveal the change in membrane roughness, providing an image at nanometer-scale resolution. The qualitative correlation between cell membrane roughness and oxidative stress level was disclosed, revealing that roughness increased with the increase in oxidative stress level.

In our study, a strong influence of TiO_2_-NPs on the changes in CHO cell membrane morphology was confirmed by changes in the roughness of the membrane and the formation of pits in the membrane. The study showed that a dose–response relationship described the change in morphology on the surface of CHO cell membranes as a function of the level of exposure to TiO_2_-NPs after 60 min. Quantifying the reaction after different exposure times revealed different relationships and possibly different conclusions regarding the effect of the nanoparticles in question. Even if the result of the study was a qualitative indication of the effect of TiO_2_ NPs, it opens the way for statistical quantitative correlations.

## 4. Conclusions

The interaction between TiO_2_-NPs and the CHO cell membrane was investigated using AFM and Raman spectroscopy. The main conclusions based on the research results and analysis are as follows:

The interaction of TiO_2_-NPs produced pits in the cell membrane and increased the membrane roughness with exposure time.

The depth of the pits varied in the range of 40–80 nm. It is assumed that these pits were caveolae participating in the endocytosis of TiO_2_-NPs.

The changes in the CHO cell membrane Raman spectra after exposure to TiO_2_ nanoparticles demonstrated the role of proteins in the interaction mechanisms between nanoparticles and the CHO cell membrane.

The determined changes in the CHO plasma membrane morphology after exposure to TiO_2_-NPs suggested that TiO_2_-NPs could affect cell physiology or even cell death. Atomic force microscopy integrated with Raman spectroscopy was shown to be a promising approach for the investigation of mechanisms via which nanoparticles interact with cell membranes.

This work enhances the current knowledge of the interaction of TiO_2_ nanoparticles with mammalian cells and furthers the understanding of the nanotoxicity and nanoparticle transport mechanism across membranes.

## Figures and Tables

**Table 1:** XRD results (Rietveld).

Phase Name	Crystallite Size	Strain
Rutile, syn	60.3 (3)	0.0004 (13)

**Table 2:** Quantitative analysis of the results (RIR).

Phase Name	Content (%)
Rutile, syn	100.0 (10)

**Table 3:** Data sheet from manufacturer (Nanostructured & Amorphous Materials Inc., Los Alamos, NM, USA).

Product 5486 WJ Titanium Oxide	Purity	Average Particle Size	Specific Surface Area	Bulk Density
TiO_2_ rutile	99.8%	60 nm	20–40 m^2^/g	0.49 g/mL

## Data Availability

The data presented in this study are available on request from the corresponding author.
